# Royal Westminster Ophthalmic Hospital

**Published:** 1829-11

**Authors:** 


### ROYAL WESTMINSTER OPHTHALMIC HOSPITAL.

*Cases reported by* Mr. Foote.

### *Chronic Catarrhal Inflammation, with Specks on the Cornea.*

Ellen Morvyn, æt. eight. December 27th, 1828. Her present affection is attributed to a cold she contracted in the beginning of the winter.—Applic. Ung. Arg. Nitr. Pulv. Alter. i. omni nocte et mane sumend.

30th.—Better. Rep. medic. et applic. unguent.

January 1st.—Is a great deal better. Rep. ung.; pulv. rep.

6th.—Discharged cured.

### *Pustular Inflammation.*

Charles Wheeler, æt. ten. January 15th, 1829. His eyes have been inflamed five weeks. Does not complain of any pain; much lachrymation; the eyes are closed in the morning; conjunctivæ of lids and ball inflamed; a pustule on the cornea of the right eye, tending to ulceration.—Applic. Ung. Argent. Nitrat. Pulv. Jalap. C. ℈ij. mane sumend.

17th.—The left eye is nearly well; the right rather better.—Ung. repet. et Pulv.

20th.—The right eye is much the same.—Unguent. ad dextr.; Pulv. repet.

22d.—The right eye is much better: the pustule no longer threatens ulceration. The left eye is cured.—Ung. Argent. Nitr. ad dextr. tantum.

27th.—A small speck is left on the corner of the right eye.—Ung. repet. et Pulv.

29th and 31st.—Ung. repet.

February 3d.—The speck still remains. Discharged, with directions to use the Gutt. Arg. Nitr. gr. vj. ad ℥i.

*Inflammation of Conjunctiva, with Pustule of Cornea.*

Ellen Guindley, æt. two. March 7th, 1829. Disease apparently caused by the irritation of teething. Has been affected in this way for six months. Intolerance of light, with increased lachrymation, exist in a great degree.—Appl. Ung. Nigr. Pulv. Jalap. C. ℈i. mane sumend.

10th.—Child much better. Repet. Ung.

12th.—Cured. Discharged.

*Inflammation of the Conjunctiva and Sclerotic Coats, with Ulcer on the Cornea.*

Joseph Delafons, æt. eleven. May 19, 1829.

The Ung. Nigr. was applied on the 19th for a simple inflammation of the conjunctiva; he then stayed away for a week. When he returned, there was acute inflammation of the conjunctiva and sclerotic coats, with a large ulcer on the cornea, which was highly vascular. Cornea appeared rather dull, a zone of vessels surrounding it. Complained of great pain and mistiness of vision.—Appl. Ung. Argent. Nitr.

28th.—Much better. Repet. Ung.

June 4th.—Repet. Ung.

9th.—Ulcer cicatrising. Repet. Ung.

18th.—Discharged, cured.

*Wound of the Cornea; Protrusion of the Iris.*

William Barkham, ætat. twenty. December 27th, 1828. The instrument he was using, while working at his trade, slipped, and struck him in the left eye. The wound is rather large, at the inferior and inner parts of the cornea; the iris protruding.

28th.—Has not any pain. Hydr. Subm. gr. vj. h. s. s.; Magn. Sulph. ℥i. mane sumend.

29th.—Complains now of great pain. Venesectio ad ℥xiv. Pil. rep. et Magn. Sulph.

30th.—Free from pain.—A bandage to be applied with pads on the eye, keeping it closed.

This bandage was continued until the 13th of January, occasionally taking aperient medicines, when he complained of severe pain in the eye.—Cucurb. cruent. ad ℥xij. temp. sinistr. Hydr. Subm. gr. vj. h. s. s. Magn. Sulph. ℥i. mane sumend.

17th.—The cupping has succeeded in removing the pain. The bandage to be reapplied.

February 3d.—Discharged, cured; returned thanks. The iris has formed adhesions with the cornea; the pupil is irregular, being nearly oval: he says, however, that his sight is as good as ever.

---

### *Case of Iritis of both Eyes, successfully treated with the Oleum Terebinthinæ. Reported by* Mr. Weight.

Mary Ann Berry, ætat. twenty-four; admitted August 11th, 1829, with iritis of both eyes.

The inflammation commenced a fortnight ago, accompanied by aching pains in the ball of the eye. Since the first four or five days she has not observed any increase of the redness, which was a little diminished by the application of some leeches three days ago. The aching pains, though not always present, frequently occur, especially on any exposure to light: they are, however, not very violent, and do not last long. At present there is not much inflammation of the sclerotic in either eye, but it is greater in the left, forming a zone, of a reddish pink colour, round the cornea. In this eye the anterior chamber is cloudy, and the pupil of an oval shape. In the right eye, the anterior chamber has its natural transparency, but the pupil is more irregular than the left. The inflammation of the conjunctiva in both eyes is very trifling, and there is little or no increased lachrymation; her sight, she says, is as good as ever. Three days before her eyes became inflamed, an eruption broke out all over her body; little of which is now visible, but what there is looks syphilitic. She admits having had a sore throat a short time ago, but denies ever having had, to her knowledge, any of the primary symptoms of syphilis.—Ol. Terebinth. ʒi. ter quotidie.

12th.—She has had great pain in making water, and her eyes are rather more inflamed, but not painful; the bowels rather confined.—Omitte Ol. Terebinth. Capiat Pulv. Jalap. comp. ʒi. statim. Inf. Lini pro potu ordinario.

13th.—The left eye rather more inflamed, the right a little improved; the strangury continues, but is not very severe; the bowels are open.—Cont. Ol. Terebinth. ʒi. ter die.

14th.—Complains of a drawing pain in the left eye, as if a blister was applied to it, but it is not so severe now as it was during the night. The sclerotica and conjunctiva are much more inflamed, and the anterior chamber more cloudy: the pupil, however, can be distinctly seen, and is rather more circular. The pupil of the right eye is still very irregular, but the sclerotic inflammation has almost disappeared. She does not experience much pain in the urinary organs from the medicine. The bowels rather confined, and she feels very sick.—Omitt. Ol. Terebinth. Capiat Pulv. Jalap. comp. ʒi. statim.

15th.—Better. Pupil of the right eye is more regular, and sclerotic inflammation less. Pain in the left eye not so great; the pupil is more circular, and the anterior chamber is much clearer, and she can see better; the sclerotic inflammation diminished. She complains now of very little pain in making water; bowels open.—Rep. Ol. Terebinth. ʒi. ter die.

10th.—She suffered great pain in both eyes last night, but the inflammation is diminished; the right eye is nearly well. She only took one dose of Ol. Terebinth. yesterday. There is no strangury, but she feels very sick.—Cont. Ol. Terebinth. Emp. Belladonn. tempor.

17th.—The pain a little relieved, but still severe, with great intolerance of light; the anterior chamber of the left eye much clearer, the conjunctiva rather more inflamed; the pupil of the right eye is slightly irregular, and the vessels round the edge of the cornea still remain injected.—Applicentur Hirudines sex temp. sinistro. Cont. Ol. Terebinth.

20th.—She can see much better, but the anterior chamber is still dim; the pupil regular. She took only one dose of medicine yesterday.—Cont. Ol. Terebinth. ter die.

24th.—Has not taken any medicine since the 20th. The inflammation, however, is considerably diminished; the iris is less discoloured; the pain continues, but not so severe.—Cont. Ol. Terebinth. ter die.

25th.—The eye rather more painful; the pupil a little irregular. The medicine produces giddiness, and slight pain in making water. —Magnes. Sulphat. ℥i. mane sumend. Cont. Ol. Terebinth.

29th.—She has taken her medicine regularly since the 25th. She cannot see so well to-day, and she complains of having frequent flashes of light before her eyes in the course of the day. There is a slight redness of the sclerotic of the right eye.—Cucurbit. cum ferro ad ℥x. Capiat. Ol. Terebinth. ʒiss. ter die.

September 1st.—The sclerotic inflammation of the left eye nearly gone; the pupil remains irregular; the right eye rather more inflamed. The medicine produces giddiness, but no scalding.—Pulv. Jalapæ C. ʒi. mane. Cont. Ol. Terebinth.

5th.—The left eye is now quite well; the right is still a little inflamed, the conjunctivæ of the lids rather more vascular than usual. She complains to-day of a little scalding.—Cont. Oleum Terebinthinæ.

8th.—The pupil of the left eye remains rather irregular, and the vessels of the sclerotic slightly injected; the pupil of the right eye dilated and irregular.—Rep. Ol. Terebinth. ʒi. bis in die.

15th.—The pupils of both eyes still remain slightly irregular, and there is some inflammation of the conjunctiva of the lids, for which she was ordered the application of the nitrate of silver ointment.

17th.—The inflammation of the lids much diminished. The pupils are now regular, and redness of sclerotic quite gone.—Rep. Ung. Argent. Nitrat.

